# Microencapsulation of Anthocyanin Extracted from Purple Flesh Cultivated Potatoes by Spray Drying and Its Effects on In Vitro Gastrointestinal Digestion

**DOI:** 10.3390/molecules25030722

**Published:** 2020-02-07

**Authors:** Cristina Vergara, María Teresa Pino, Olga Zamora, Javier Parada, Ricardo Pérez, Marco Uribe, Julio Kalazich

**Affiliations:** 1 Instituto de Investigaciones Agropecuarias, INIA La Platina, Santa Rosa 11610,8831314, Santiago, Chile; cristina.vergara@inia.cl (C.V.); olga.zamora@inia.cl (O.Z.); jrperezd@gmail.com (R.P.); 2 Instituto de Ciencia y Tecnología de Alimentos (ICYTAL), Facultad de Ciencias Agrarias, Universidad Austral de Chile, Av. Julio Sarrazin s/n Campus Isla Teja 5090000, Valdivia, Chile; javier.parada@uach.cl; 3 Instituto de Investigaciones Agropecuarias, INIA Remehue, Ruta 5 Sur, kilometro 8 5290000, Osorno, Chile; muribe@inia.cl (M.U.); jkalazic11@gmail.com (J.K.)

**Keywords:** encapsulation efficiency, anthocyanins, purple flesh cultivated potato, *Solanum tuberosum*, bioaccesibility

## Abstract

Purple flesh cultivated potato (PP) is a foodstuff scarcely cultivated in the world but with high potential because of its anthocyanin content. Moreover, it has been little explored as a source of anthocyanins (AT) for further applications in formulated food products. The main goal of this research was to study the effect of maltodextrin (MD) and spray drying conditions on the encapsulation efficiency (EE) and bioaccesibility of AT from purple flesh cultivated potato extract (PPE). The anthocyanin-rich extract was obtained from PP and microencapsulated by spray-drying, using MD as the encapsulating agent. A statistical optimization approach was used to obtain optimal microencapsulation conditions. The PPE microparticles obtained under optimal conditions showed 86% of EE. The protector effect of microencapsulation on AT was observed to be stable during storage and in vitro digestion. The AT degradation rate constant was significantly lower for the PPE-MD than for the PPE. The assessed bioaccesibility of AT from the PPE-MD was 20% higher than that of the PPE, which could be explained by the protective effect of encapsulation against environmental conditions. In conclusion, microencapsulation is an effective strategy to protect AT from PP, suggesting that AT may be an alternative as a stable colorant for use in the food industry.

## 1. Introduction

Consumer trends show an increasing interest in replacing synthetic dyes with natural colorants, mainly because synthetic colorants have been restricted by the official regulations of the EU and the USA due to their possible adverse effects on human health [1,2]. Thus, the identification and study of new raw materials as sources of natural colors is a relevant issue for the food industry. In this regard, flesh colored potato (*Solanum tuberosum* L.) are a potential source to obtain natural colors, besides, they are a good source of carbohydrates, proteins, dietary fiber, minerals, vitamins (vitamin C), and polyphenols [3,4]. In addition, other compounds with functionality such as high-amylose starch and short peptides have been reported [5,6].

The purple flesh cultivated potato (PP) (*Solanum tuberosum* L.) obtained by conventional breeding could be a rich source of phenolic compounds and anthocyanins, depending on its stability and availability. Anthocyanins (AT) are the most important group of water-soluble pigments in plants and are responsible for red, purple, and blue colors [7,8,9]. Also, AT have been associated with antioxidant and anti-inflammatory properties, suggesting that they may have the potential to prevent obesity and ameliorate insulin-resistance and diabetes [10,11]. Specifically, cultivated potato ATs have been associated with cancer development inhibition [12,13]. In sweet potatoes and cultivated potatoes with purple flesh, the main anthocyanins are those with acylated structures, including delphinidin, petunidin, and malvidin derivatives, showing different anthocyanin profiles and starch structures [14,15,16].

Natural colorant stability and potential biological activity are an important issue to consider for AT’s use as a colorant and/or ingredient for healthy foods. Such AT stability could be highly affected by environment (temperature, pH and light), food (pH, enzymes and other food components) and gastrointestinal conditions (pH and enzymes), limiting the application of ATs as food ingredients [17,18]. These problems, which can affect AT as before as after of ingestion, could be overcome by encapsulation technology [17,18,19,20,21]. Microencapsulation is a technique wherein a bioactive compound is encapsulated by a biopolymer, in order to protect the compound from oxygen, water, or other conditions, thereby improving its stability and release in desired stage [17,19,21]. Spray-drying is a common method used for encapsulation of bioactive compounds as anthocyanins [17,18,21,22,23,24,25,26] because of its short drying time (5–30 s), relatively low cost, and ability to change liquid solutions into powders for easier handling and greater stability [19,27]. However, it is considered an immobilization technology rather than a true encapsulation technology, because some active compounds may be exposed superficially on the microparticle [28]. Thus, the encapsulation efficiency is a way to gauge successful AT-encapsulation which should result in a powder that has minimum surface AT content on the microparticles [17].

There is no reported information on AT encapsulation for purple cultivated potato. This is the first microencapsulation study focused on the encapsulation efficiency and bioaccesibility of AT from purple flesh cultivated potato. ATs from others sources have been encapsulated, such as those from sweet potato (*Ipomoea batatas* L.) [29,30], blueberry (*Vaccinium* spp.) [9,21,23], maqui (*Aristotelia chilensis* (Mol.) Stuntz) [18,24], purple corn (*Zea may* L.) [9], purple rice (*Oryza sativa* L.) [25], black-carrot (*Daucus carota* L.) [31], and other berries [32]. Studies on AT microencapsulation have focused mainly on evaluating AT protection and stability during storage [9,22,23,25,29,31]. Currently, some studies about the evaluation of in vitro-simulated AT digestion have been reported [18,21,24,27], because this topic is relevant to establish the application and use of AT microparticles as a food ingredient. In this context, the encapsulating agent (AE) is essential in the encapsulation process to ensure the protection, stability, and release of ATs in food and during their passage through the gastrointestinal tract. AT encapsulation has been reported using maltodextrin DE 20 [22,24], inulin and sodium alginate [18], a mixture of maltodextrin DE 20 and hi-maize [21], and carboxymethyl starch/xanthan gum combinations [23]. In this particular study, maltodextrin (MD) was selected as the encapsulating agent, due to its high solubility in water, low viscosity, bland flavor, and colorlessness [33].

The main goal of this work was to study the effect of maltodextrin (MD) and spray drying conditions on the encapsulation efficiency (EE) and bioaccesibility of AT from purple flesh cultivated potato extract (PPE). 

## 2. Results

### 2.1. Characterization of Raw Material and PPE

The physico-chemical characterization of the purple flesh cultivated potato (PP) as a raw material and as its extract (PPE) is shown in Table 1.

The HPLC profile of the anthocyanins from PP showed two anthocyanins, delphinidin (61.5%) and peonidin (38.5%), and their derivatives. According to the literature, certain types of AT have been associated with purple/red cultivated potatoes. These types include conjugates of peonidin and acylated delphinidin [16,34], while malvidin and petunidin derivatives can be found in purple/blue-fleshed potatoes [35]. Differences in the AT content and composition between cultivars are explained by genetic differences and their geographical origins (for example, ssp. tuberosum vs. ssp. andigena).

The results for PPE show the concentrations of soluble solids, AT content, and FRAP, according to the use of water extract evaporation.

### 2.2. The Encapsulation of Anthocyanins from PPE

In this study, a composite central design optimized by response surface methodology (RSM) was applied to study the optimal conditions for the microencapsulation of PPE with MD by spray-drying (SD). The independent variables were the inlet air temperature (process variable) and the PPE:MD ratio (formulation variable) on the anthocyanins’ EE and Yield. RSM was applied to maximize the response variables, and the Desirability Function (DF) was applied to optimize the encapsulation of PPE. Table 2 shows the experimental runs and their response variables for the microencapsulation of PPE by spray-drying.

The EE of AT ranged from 59.4% to 93.2%. The analysis of variance (ANOVA) for the EE of the anthocyanins showed that the EE of ATs was significantly affected by the linear form (*p* < 0.05) of the inlet air temperature and the PPE:MD ratio, as well as by the quadratic form (*p* < 0.05) of the PPE:MD ratio. This model explained over 95% of the variability (R^2^ 92.9% and R^2^adj. for d.f. 90.3%), with residual values below 6.0.

Moreover, the lack-of-fit was not significant, indicating that the mathematical model fits well with the experimental data. The equation describing the effect of the independent variables on the EE of PPE is the following:

(1) $$EE\left(\%\right)=51.6407-0.0711801\;X_{1}+29.9314\;X_{2}-4.71083\;X_{2}^{2}$$

**Figure 1:**
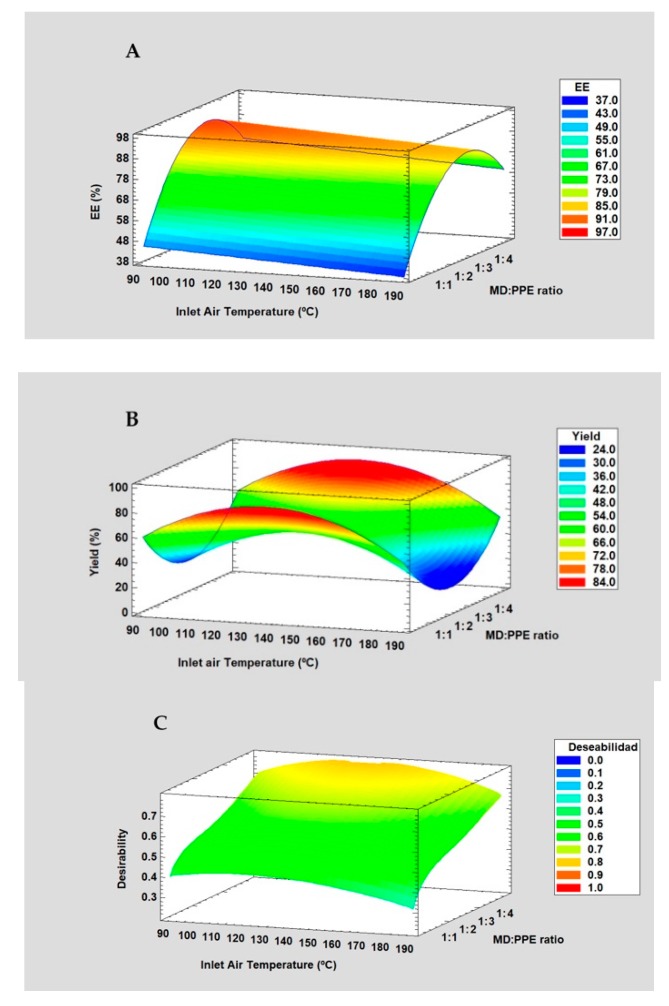
RSM plot for the EE of AT (**A)**, the yield (**B**), and multiple optimization applying DF (**C**).

The RSM plot is shown in Figure 1A. For the PPE:MD ratio, the EE of AT increased when MD content increased, which can be attributed to the rapid dry crust formation over the droplets’ surfaces [36], whereas the inlet air temperature had only a slight influence on the EE of AT because it’s not significantly factor.

The Yield of the process (powder recovered in spray-drying) ranged from 10% to 81%. Similar yield was reported for maqui juice microparticles (64.1%) using the same spray-drier. The ANOVA for the yield of the PPE-MD system showed that the linear form (*p* < 0.05) and quadratic form (*p* < 0.05) of the inlet air temperature and PPE:MD ratio had a significant effect on the Yield. The models explained over 95% of the variability (R^2^ 83.4% and R^2^ adj. for d.f. 83.3%), with residual values below 6.0. Moreover, the lack-of-fit was not significant, indicating that the mathematical model fits well with the experimental data. The equation describing the effect of the independent variables on the yield of the process is the following:

(2) $$Yield\left(\%\right)=-168.437+3.77964\;X_{1}-28.8586\;X_{2}-0.0138219\;X_{1}^{2}+5.91072\;X_{2}^{2}$$

The RSM plot is shown in Figure 1B. The yield was higher with a higher MD content, whereas the inlet air temperature had an effect at medium temperature values.

For both independent variables the major effect was attributed at PPE:MD ratio. The EE of AT and the yield of the process (response variables) were maximized, and the RSM plot is shown in Figure 1C. The optimal inlet air temperature was 130 °C, whereas the optimal PEE:MD ratio was 1:4. Thus, the response variables were maximized at medium values of inlet air temperature (°C) and a high MD content (both within the range studied).

### 2.3. Characterization of the PPE-MD System Obtained Under Optimal Conditions

Table 3 shows the physical and chemical characteristics of the PPE-MD system obtained under optimal conditions. The optimal conditions (inlet air temperature and PPE:MD ratio) of microencapsulation by spray-drying were 130 °C and 1:4, respectively. The EE correspond to AT-Encapsulating agent (MD) interaction. The EE of AT obtained in this study was 86%. This result can be explained by the high AT-MD interactions caused by hydrogen bonding and/or electrostatic interactions [24]. Similar EE of AT were reported for blueberry and pomegranate microparticles using MD 74.4–85.2% [21] and 89.4–100% [37], respectively. Fredes et al. (2018) [18] reported EE value of 92.5% for maqui juice microencapsulated with MD. The AT recovery is the parameter that demonstrates the effect of spray-drying process on AT content (or stability during spray-drying). The AT recovery in the PPE-MD was over 95%, showing the effect of the short exposure time to temperature and rapid crust formation. Our result is in agreement with other studies where obtaining AT microparticles by SD with MD, for maqui juice reported recovery of 99,8% [18], for pomegranate extract and juice recovery of 97% and 100% [37] and for bayberry extract recovery of 94% [38]. The moisture, aw, hygroscopicity, and particle size were within the range described for anthocyanin microparticles obtained by spray-drying with MD [18,26,36,39].

**Figure 2:**
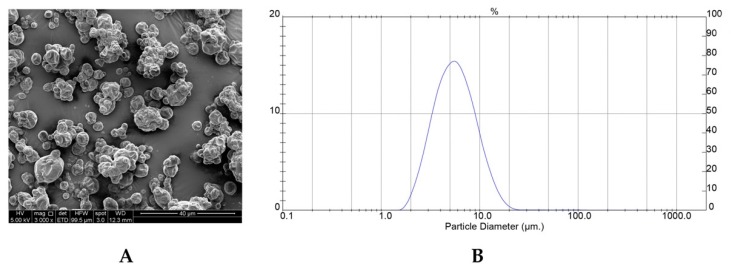
Scanning electron microscopic (SEM) photograph for PPE-MD (S) (**A**) and Particle size distribution (**B**).

Figure 2A,B shows the SEM photographs and particle size distribution of PPE-MD obtained under optimal conditions. The surface morphology of the PPE-MD system shows irregular, spherical shapes with some shrinkage and agglomeration tendencies. The formation of the indented surfaces found on particles during spray-drying is attributed to particle shrinkage that can take place both at high and low inlet air temperatures. At high inlet temperatures, the rapid water evaporation and high pressure inside the particles produced shrinkage, whereas at low temperatures (as in the case of PPE-MD systems), water diffusion was slower, and the particles took longer to shrink [40]. A similar external morphology was observed in particles of anthocyanin pigment encapsulated with MD, the AT from black carrot [31], pomegranate extract [37], maqui juice [18,24], and sweet potato [22].

The distribution of particle sizes was unimodal with a normal distribution (Figure 2B), with a range between 1.95 and 20.9 µm, and a span index of 1.36 (polydispersity), which are typical for the type of atomizer used (double-fluid for SD) [41].

### 2.4. Stability of Microencapsulated PPE during Storage

**Figure 3:**
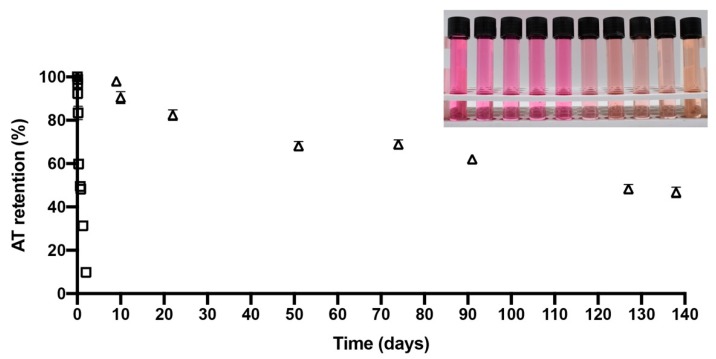
Evolution of anthocyanin retention from PPE and PPE-MD during storage at 60 °C. (PPE (☐) and PPE-MD (Δ). The photograph in Figure 3 shows the visual degradation of AT (evolution in color degradation) on analysis solution at during the time-course of the storage stability assay.

Figure 3 shows an AT evolution retention versus time line (days) for PPE and PPE-MD systems during storage at 60 °C. The PPE-MD system (encapsulated) showed the highest AT retention over time (45% at 138 days after storage at 60 °C). In contrast, the PPE (non-encapsulated) decreased its AT retention to 10% after 2 days in storage. The rapid (two day) AT degradation in PPE shows the importance of the encapsulating agent in the degradation of AT from purple potatoes. The AT storage stability was carried out for microparticles in powder (PPE-MD) and liquid non-encapsulated PPE. The photograph in Figure 3 shows the visual degradation of AT (evolution in color degradation) for liquid (analysis solution) non-encapsulated PPE during the time-course of the storage stability assay.

The degradation kinetics of AT followed pseudo-first order behavior for PPE and PPE-MD systems. The same order has been reported for the AT degradation from maqui juice [24], black carrot extract [31], pomegranate juice extract [37], and red flower cabbage [42]. AT degradation is mainly caused by oxidation, cleavage, or oxidation caused by heating [43].

Table 4 shows the AT degradation rate constant at 60 °C for the PPE (non-encapsulated) and PPE-MD systems (encapsulated), as well as the color difference values (ΔΕ) of both systems. The AT degradation rate constant (k_obs_) was obtained from the slope of the logarithmic plots of the percentage retention vs. time (days). The correlation coefficient (r^2^) was higher than 0.97, indicating a good data fit. Thus, the AT degradation rate constant of the PPE-MD system (0.53 × 10^2^ days^−1^) was significantly lower (*p* < 0.05) than that of PPE (11.18 × 10^2^ days^−1^). A similar tendency is observed for ΔΕ.

The evolution of the AT retention versus time (days), the AT degradation rate constant at 60 °C, and ΔΕ showing the protection of encapsulation on the degradation of AT. The encapsulation improved AT stability due the AT-MD interactions (attributed to AT-encapsulating agent interaction that may occur by electrostatic interactions or hydrogen bonding [18,24]), which reduced the damage of active AT caused by adverse storage conditions, thereby extending shelf life and color. These results are in agreement with the previous report on AT encapsulation obtained for black-carrot [31], pomegranate [37], black berry [44], maqui [18,24], plum [39], and blueberry [21], in which microencapsulation techniques significantly improved the stability of AT and phenolic compounds.

### 2.5. In Vitro Bioaccessibility of Anthocyanins

Bioaccessibility (BA) has been defined as the amount of compound that is released from the matrix after digestion [45]. Table 5 shows the final BA (%) of AT after the in vitro digestion of PPE (non-encapsulated) and PPE-MD (encapsulated).

The results show that encapsulation significantly increased the observed BA of AT, with the final BA of PPE-MD being ~45% higher than PPE (see Table 5). Since in PPE the AT are basically free from any matrix—which can be understood as a 100% bioaccessibility of AT after the start of digestion—the lower values of BA are possibly due to the loss of these molecules by environmental condition. On the other hand, encapsulation appears to protect the AT, during digestion, against environmental conditions (especially pH). The AT release from PPE-MD in the gastric digestion fraction corresponds to the superficial AT (on the microparticle). The final BA of the PPE and PPE-MD systems was higher than that described by Fredes et al. (2018), for free and encapsulated maqui juice, and by da Rosa et al. (2019), for free and encapsulated blueberry extract.

## 3. Materials and Methods

### 3.1. Raw Materials

Purple potato extract (PPE) was obtained from a PP advanced breeding line (*Solanum tuberosum* L.) belonging to the potato breeding program for the flesh color of potatoes at the Agricultural Research Institute of Chile (INIA). This purple flesh cultivated potato was cultivated in the south of Chile (40° 31′ 18.44” S; 73° 03′ 47.01” W). A pool of tubers was selected in the field completely at random according to the experimental design.

The maltodextrin (MD) (DE = 20) (Prinal, Santiago, Chile) was used as encapsulating agent.

### 3.2. Preparation of the Purple Potato Extract (PPE)

A pool of PP tubers was washed with a cold-water spurt and grounded in a blender (Thermomix, Vorwek, Germany) to obtain a puree according to Wang and Xu (2007) [44] with modifications. Briefly, pH was adjusted (pH 3) to inactivate oxidation and then PPE was extracted by pressing. The PPE was centrifuged, and the AT-rich fraction was concentrated in a rotary evaporator R-100 (Büchi, Flawil, Switzerland) at 50 °C until reaching 65° Brix.

#### 3.2.1. Characterization of PPE

The PPE was evaluated in term of its moisture content by an infrared moisture analyzer (PMB202, ADAM, Maidstone Road, UK), soluble solids (° Brix) with a refractometer (HI 96801, Hanna instruments, Rhode Island, USA), and the pH was measured with a pH-meter (UB-10, Denver instrument, Colorado, USA). All the analyses were carried out in triplicate.

#### 3.2.2. AT content and HPLC Profile

Total anthocyanins: The total anthocyanins were determined by the pH differential method according Lee et al. (2005) [46] by spectrophotometry (spectrophotometer V-700, Jasco, Tokyo, Japan). All analyses were carried out in triplicate.

Anthocyanin profile by HPLC-DAD: Anthocyanin analysis was done in a chromatographic system HPLC consisting of a Jasco interface LC-NetII/ADC with a diode array detector MD-4010 and an autosampler AS-4050 (Jasco, Tokyo, Japan) with a C18 column (Kromasil-100, 3.5 mm i.d × 150 mm). The samples (20 μL) were then injected. The mobile phases, A (0.2% *v/v* TFA in water), B (0.2% *v/v* TFA in acetonitrile), and C (0.2% *v/v* TFA in methanol), were used under the following conditions: initial, 8% B and C maintained for 5 min; an 11 min linear change to 12% B and 7% C; a 12 min linear change to 11.5% B and C; a 20 min linear change to 13.5% B and C; a 25 min linear change to 20% B and C; a 30 min linear change to 50% B and C.

### 3.3. Microencapsulation of PPE

#### 3.3.1. Preparation and Characterization of PPE Microparticles

PPE microencapsulated with MD was prepared as follows: MD (5–20 g) was dissolved in distilled water and heated at 70 °C. The infeed solution (100 g) was elaborated according to the experimental design (Table 1) and mixed with PPE (5 g) via constant stirring. The resulting solutions were homogenized at 11,000 rpm for 3 min with a Polytron PT 2100 (Kinematica A.G, Luzern, Switzerland) and fed into a B-290 mini spray-dryer (Büchi, Flawil, Switzerland). The spray-dryer operated at the inlet air temperature (Table 1), with an air flow of 600 L/h, a rate of feeding of 3 mL/min (5%), and an atomization pressure of 5 bar. The powders obtained were stored in the dark and kept at −20 °C for subsequent analysis. The encapsulating agent maltodextrin (MD) was obtained from National Starch and Chemical, S.A., Chile.

Moisture and anthocyanin content were determined for microparticle characterization. Water activity (aw) was determined by the dewpoint method (Hygrolab 2, Rotronic Instrument Corp, Hauppauge, New York, NY, USA) at 20 ± 0.3 °C. Hygroscopicity was determined at 99% of the relative humidity according to Cai and Corke (2000) [47].

#### 3.3.2. Morphology

The outer structures of the microencapsulated PPE were examined by scanning electron microscopy (SEM). The powder was coated with gold/palladium using a Varian PS 10E vacuum evaporator and analyzed using a LEO 1420VP SEM (LEO Electron Microscopy Ltd., Cambrige, UK) operating at 20 kV. The images were then collected digitally (EDS 7424 software, Oxford Instruments, Oxford, UK).

#### 3.3.3. Particle size

The particle size and size distribution were determined by laser light scattering using Mastersizer X (Worcestershire, Malvern Instrument, Worcestershire, UK).

The powder was dispersed in recirculating propilenglycol, and the mean particle size was expressed as the volume mean diameter (D4,3). The polydispersity was given by the span index, which was calculated according to Equation (3) [41].

(3) $$Span=\frac{D\left(0.9\right)-D\left(0.1\right)}{D\left(0.5\right)}$$

where D0.1, D0.5, and D0.9 correspond to the diameters relative to 10%, 50%, and 90% of the accumulated size distribution.

#### 3.3.4. Encapsulation efficiency of the AT and the yield of the process

The EE and Yield were calculated according to Equations (4) and (5), respectively.

(4) $$EE\left(\%\right)=\frac{Experimental\;total\;anthocyanins-Superficial\;anthocyanins}{Experimental\;total\;anthocyanins}\times 100$$

(5) $$Yield\left(\%\right)=\frac{Powder\;after\;spray\;drying\left(g\right)}{Solid\;in\;the\;feed\;solution\;\left(g\right)}\times 100$$

Experimental total anthocyanin determination: the microparticles was dissolved by the following procedure: Microparticles (200 mg) were dispersed in 2 mL of methanol:acetic acid:water (50:8:42 *v*/*v*/*v*) and stirred using a vortex mixer for 1 min. Then, they were ultrasonicated twice for 10 min and finally centrifuged at 112,000 rpm for 5 min [48]. The anthocyanin content was determined by the pH differential method.

Superficial anthocyanin determination: Microparticles (100 mg) were dispersed in a 2 mL ethanol:methanol (1:1) solution, stirred in a vortex mixer for 1 min, and centrifuged at 112,000 rpm for 5 min [48]. The anthocyanin content was determined by the pH differential method.

#### 3.3.5. Accelerated Storage Stability Test of the Microparticle Powders

Storage stability test: Microparticle (PPE-MD) powders and PPE (non-encapsulated) (100 mg) were transferred to clear glass vials (16 × 100 mm) and stored at 60 ± 1 °C in a forced-air oven (UFE 500, Memmert, Schwabach, Germany) with a controlled temperature and in the absence of light. To determine the anthocyanins’ kinetic degradation, the study was performed over four months for the PPE-MD microparticles and for 48 h for the PPE (non-encapsulated). Triplicate vials were withdrawn at specific times to determine AT content.

Kinetic analysis. The data were best fit by a first-order kinetic model according to Equation (6):

(6) $$LnC=LnC_{0}-kt$$

where C_0_ is the initial concentration of anthocyanin (mg anthocyanin/g), C is the anthocyanin concentration at time t, k is the degradation rate constant, and t is the storage time. The degradation rate constants (k) and correlation coefficient were obtained from the slope of a plot of the natural log of the percentage retention of anthocyanin vs. the time for the first order at each studied temperature (60 °C).

Color difference value (ΔE): ΔE was defined as Equation (7):

(7) $$∆E=\sqrt{{\left(L^{*}-L\right)}^{2}+{\left(a^{*}-a\right)}^{2}+{\left(b^{*}-b\right)}^{2}}$$

where L*, a*, and b* are the values of the samples at zero time and L, a, and b are the measured values of each sample at the final time.

The color parameters, L*, a*, b* were determined by a colorimeter (Ultrascan pro, Hunter Lab, Reston, VA, USA).

#### 3.3.6. In vitro digestion model

In vitro digestion was performed according to Aravena et al.’s [43] protocol with modifications.

##### Mouth digestion

Briefly, PPE microparticles and PPE (non-encapsulated) samples (0.5 g) were weighed; then, Artificial saliva (9 mL) was added to each flask with a sample. This solution was comprised of 14.4 mM sodium bicarbonate, 21.1 mM potassium chloride, 1.59 mM calcium chloride, and 0.2 mM magnesium chloride. The pH was adjusted to 7 with HCl (1 M). Sixty α-amylase units per milliliter of buffer were incorporated the same day the test was performed. Samples were incubated in a thermostatic bath (ZHWY-110 × 30, Zhicheng, Shanghai, China) at 37 °C for 5 min, with a shaking speed of 185 rpm.

##### Gastric digestion

The pH of the samples was adjusted to 2.0 using HCl (1 M); then 36 mL of a pepsin solution (25 mg/mL in 0.02M HCl) was added. In this way, each sample was diluted 5-fold with pepsin solution. Samples were incubated for 2 h at 37 °C with a stirring speed of 130 rpm.

##### Gut digestion

The pH of the samples was adjusted to 6.0 with NaHCO3 (1 M). Then, 0.25 mL (per mL of the sample) of an artificial gut solution, containing pancreatin (2 g/L) and bile salts (12 g/L) dissolved in aqueous NaHCO3 (0.1 M), was added. Incubation was carried out for 2 h at 37 °C with a shaking speed of 45 rpm. Each digestion product was transferred to 50 mL Falcon tubes, and the pH was adjusted (pH 3) [49]. The solution was then centrifuged for 10 min at 5000 rpm to recover the liquid fraction. Next, the liquid digestion product was centrifuged at 12,000 rpm before anthocyanin analysis. The measured number of anthocyanins was the bioaccessible portion, which was calculated according Equation (8):

(8) $$Global\;bioaccessibility\;\left(Global\;BA\%\right)=\frac{mg\;anthocyanin\;in\;digest\;product}{mg\;anthocyanin\;in\;PPE\;or\;\left(PPE-MD\right)}\;\times \;100$$

#### 3.3.7. Experimental Design

For purple flesh cultivated potato in the field, the experiment used a randomized block design with four replicates. The plot size for each replicate was 6 rows wide and 10 m long (20 m^2^). The four central rows were harvested for further agronomic analysis, and a randomized tuber pool per replicate was selected for chemical characterization and microencapsulation.

The encapsulation of PPE was performed by applying a central composite design (CCD) (with 12 runs: 4 experimental points, 4 axial points, and 4 central points) with two replicates. The inlet air temperature (100–180 °C) and PPE:MD ratio (1:1–1:4) were evaluated as independent variables of the encapsulation efficiency (EE) of anthocyanins and the yield of the process (%). In this study, response surface methodology (RSM) was used to find the optimal conditions via multiple response optimization using the desirability function (DF). The DF is a function that assigns a score between 0 and 1 to a set of responses and chooses the factors that maximize that score (where 1 represents a completely desirable value, such as 100% EE or yield) [50,51].

The data were fitted to a second-order regression model according to Equation (9). All of the experiments were conducted randomly to avoid systematic bias.

(9) $$Y=b_{0}+\overset{2}{\underset{i=1}{\sum }}b_{i}X_{i}+\overset{2}{\underset{i=1}{\sum }}b_{ii}X_{i}^{2}+\overset{1}{\underset{i=1}{\sum }}\overset{2}{\underset{j=i+1}{\sum }}b_{ij}X_{i}X_{j}$$

where *Y* is the response; subscripts *i* and *j* range from 1 to the number of variables (*n* = 2); *b*_0_ is the intercept term; the *b_i_* values are the linear coefficients; the *b_ij_* values are the quadratic coefficients; and *X_i_* and *X_j_* are the levels of the independent variables.

#### 3.3.8. Statistical Analysis

The differences in the raw material, PPE, PPE-MD system, and BA for each parameter were analyzed using a one-way ANOVA. When significant differences were found, a Tukey test (*p* < 0.05) was applied. Linear regression was used to determine the reaction order and the rate constants on anthocyanin stability. All statistical analyses were performed using a Statgraphics centurion XVIII software (StatPoint, Inc., Warrenton; VA, USA).

## 4. Conclusions

The encapsulation technology is a useful strategy to protect AT from purple flesh cultivated potato. This protection showed stability during storage and within the in vitro gastrointestinal digestion model, as well. Both the AT degradation rate constant and bioaccessibility were significantly higher in the PPE-MD system (encapsulated) than in PPE (non-encapsulated). These results are the first to study AT from flesh purple cultivated potatoes and contribute to the development of new purple potatoes products potentially useful as colorant or health ingredients (antioxidant and anti-inflammatory properties) in powder formulation for the food industry. The next steps are to establish the stability, bioaccessibility, and bioavailability of individual AT in order to select potato lines with specific anthocyanin profiles and to evaluate the effects of others encapsulating agents to help solve food industry challenges.

## Figures and Tables

**Table 1:** Physico-chemical characterization of Purple flesh potato and its extract (PPE).

Sample	PP	PPE
Moisture content (%)	75.6 ± 3.7 ^a^	35.0 ± 0.4 ^b^
Soluble solids (°Brix at 20 °C)	5.2 ± 0.1 ^b^	65.0 ± 0.5 ^a^
Total Anthocyanins (mg cy-3-glu/g)	0.24 ± 0.02 ^b^	2.0 ± 0.1 ^a^
Antioxidant capacity (FRAP) (mg TE/g)	4.7 ± 0.3 ^b^	21.1 ± 0.5 ^a^

PPE: purple potato extract; cy-3-glu: cyanidin-3-glucoside; TE: Trolox equivalent. Different letters indicate statistically significant differences between systems for the Tukey multiple range test (*p <* 0.05). a correspond to highest values and b correspond to lowest values.

**Table 2:** Encapsulation efficiency of anthocyanins (EE) and yield for the PPE microencapsulated by spray-drying according to the central composite design.

Factors			Response Variables	
Runs	Inlet Air Temperature (°C) [X1]	PPE:MD ratio [X2]	EE (%)	Yield (%)
1	100 (−1)	1:1 (–1)	72.8 ± 4.1	60.0 ± 4.1
2	180 (+1)	1:1 (–1)	62.3 ± 0.4	39.6 ± 0.3
3	100 (−1)	1:4 (+1)	93.2 ± 1.2	47.1 ± 1.2
4	180 (+1)	1:4 (+1)	84.7 ± 1.5	40.0 ± 0.5
5	92 (−1.21)	1:2.5 (0)	85.6 ± 0.3	20.0 ± 0.7
6	188 (+1.21)	1:2.5 (0)	85.0 ± 1.6	22.0 ± 0.2
7	140 (0)	1:0.7 (−1.21)	59.4 ± 0.4	64.7 ± 0.4
8	140 (0)	1:4.3 (+1.21)	78.8 ± 0.2	81.0 ±1.6
9	140 (0)	1:2.5 (0)	88.5 ± 0.7	57.1 ± 1.6
10	140 (0)	1:2.5 (0)	87.0 ± 1.6	56.0 ± 1.6
11	140 (0)	1:2.5 (0)	89.0 ± 1.6	50.0 ± 1.6
12	140 (0)	1:2.5 (0)	85.1 ± 1.6	56.6 ± 1.6

PPE: purple potato extract; MD: maltodextrin; EE: encapsulation efficiency.

**Table 3:** Physical and chemical characterization of PPE-microparticles obtained under optimal conditions.

System	PPE-MD
Inlet air temperature (°C)	130
PPE:MD ratio	1:4
EE (%)	86.0 ± 0.6
Yield (%)	58.9 ± 1.0
Moisture content (%)	5.6 ± 0.4
Water activity (aw)	0.225 ± 0.001
Hygroscopicity (g/100 g)	33.6 ± 2.7
Particle size (D4,3)	6.51 ± 0.1
Total Anthocyanin (mg cy-3-glu/g)	1.34 ± 0.02
Antioxidant capacity (FRAP) (mg TE/g)	10.1 ± 0.6

PPE: purple potato extract; MD: maltodextrin; EE: encapsulation efficiency; Cy-3-glu: cyanidin-3—glucoside; TE: Trolox equivalent.

**Table 4:** Anthocyanin degradation rate constant at 60 °C for PPE and PPE-MD and their color difference values.

System	k(obs) ± DS (days-1)	r^2^	ΔΕ
**PPE**	11.18 × 10^2^ ± 0.10 × 10^2 a^	0.971	58.3 ± 0.3 ^a^
**PPE-MD**	0.53 × 10^2^ ± 0.02 × 10^2 b^	0.974	18.4 ± 0.2 ^b^

PPE: purple potato extract; PPE-MD: purple potato extract encapsulated with maltodextrin; ΔΕ: color difference values. Different letters indicate statistically significant differences between systems for the Tukey multiple range test (*p* < 0.05), a correspond to highest values and b correspond to lowest values.

**Table 5:** Bioaccessibility of anthocyanins from PPE and PPE-MD after in vitro digestion.

System	Anthocyanins (mg cy-3-glu/g)			Gastric BA	Final BA (%)
	Before Digestion	After Gastric Digestion	After Intestinal Digestion	(%)	
PPE	2.010 ± 0.050	0.957 ± 0.048	0.913 ± 0.040	47.6 ± 3.5 ^b^	45.4 ± 2.3 ^b^
PPE-MD	1.340 ± 0.020	1.028 ± 0.064	0.887 ± 0.099	76.7 ± 4.2 ^a^	66.2 ± 9.1 ^a^

PPE: purple potato extract; PPE-MD: PPE encapsulated with maltodextrin; BA: bioaccessibility of anthocyanins; Different letters in the same column show significant differences between systems (*p* < 0.05), a correspond to highest values and b correspond to lowest values.

## Abreviations

ANOVA	analysis of variance
AT	antohocyanins
a_w_	water activity
BA	bioaccessibility
CCD	central composite design
Cy-3-glu	cyanidin-3-glucoside
ΔΕ	color difference values
DF	desirability function
EE	encapsulation efficiency
HPLC-DAD	high-performance liquid chromatography-photo diose array detector
MD	maltodextrin
PP	purple flesh cultivated potato
PPE	purple flesh cultivated potato extract
RSM	response surface methodology
SD	spray drying
SEM	scanning electron microscopy
TE	trolox equivalent
